# Editorial: Advancing NSCLC treatment: overcoming challenges in immune checkpoint inhibitor therapy

**DOI:** 10.3389/fphar.2026.1744580

**Published:** 2026-03-26

**Authors:** Renwang Liu, Yiming Meng, Mohamed Rahouma

**Affiliations:** 1 Department of Lung Cancer Surgery, Center of Thoracic Surgery, Tianjin Medical University General Hospital, Tianjin, China; 2 Tianjin Key Laboratory of Lung Cancer Metastasis and Tumor Microenvironment, Tianjin Lung Cancer Institute, Tianjin Medical University General Hospital, Tianjin, China; 3 Department of Central Laboratory, Cancer Hospital of Dalian University of Technology, Liaoning Cancer Hospital and Institute, Shenyang, Liaoning, China; 4 Cardiothoracic Surgery Department, Weill Cornell Medicine, New York, NY, United States; 5 Surgical Oncology Department, National Cancer Institute, Cairo University, Cairo, Egypt

**Keywords:** biomarkers, combination therapy, drug resistance, immune checkpoint inhibitor, NSCLC

## Introduction

Non-small cell lung cancer (NSCLC) is a leading global cause of cancer-related death, and immune checkpoint inhibitors (ICIs) have revolutionized its treatment by reactivating the immune system to target tumor cells (Siegel et al., 2025; Zhao et al., 2025). Despite significant improvements in patient outcomes, ICIs present several key challenges: inconsistent efficacy across populations, complex resistance mechanisms, and treatment-related toxicities (Zhao et al., 2025; Tang et al., 2025). Thus, a deeper understanding of biological mechanisms, reliable biomarkers, and tumor-immune interactions is urgently needed to optimize ICI therapy and improve survival.

In this Research Topic, we have compiled 15 studies, including case reports, real-world analyses, meta-analyses, and reviews, contributed by researchers across Asia, Europe, and North America, to address these gaps and provide actionable insights for clinical practice and future research.

## Efficacy of ICIs in NSCLC of different stages

ICIs exhibit versatility across NSCLC stages. Thus, we collected recent advances in ICI therapy across different stages of NSCLC, highlighting how immunotherapy is reshaping treatment paradigms from early-stage to advanced disease. For resectable stage IB-III NSCLC, we include the work by Huang et al., which reported that adjuvant ICIs significantly improved disease-free survival (HR = 0.82) in EGFR-negative, PD-L1-positive (1%–49%), non-squamous, and non-smoking patients, with manageable safety. For unresectable stage III NSCLC patients, induction chemoimmunotherapy (pre-consolidative radiotherapy, pre-CRT) may be a flexible option, as Guan et al. reported that it yields similar efficacy to consolidation immunotherapy (post-consolidative radiotherapy, post-CRT) and requires fewer immune cycles. For advanced NSCLC, Justeau et al. conducted a French real-world study of 4,001 patients. Results showed that patients receiving second-line nivolumab had a median overall survival (OS) of 10.2 months, with direct ICI retreatment more effective than retreatment after interposed therapies[9]. Meanwhile, Chen et al. found that bevacizumab plus chemotherapy outperformed chemoimmunotherapy (median OS: 21.6 vs. 12.63 months) in PD-L1-negative, driver-gene-negative lung adenocarcinoma.

These studies highlight the expanding role of ICIs across different NSCLC stages. Efficacy is influenced by factors such as disease stage, prior treatment, PD-L1 expression, and histology, underscoring the need for a dynamic and personalized approach to ICI therapy.

## Mechanisms, biomarkers, and safety

Identification of biomarkers and underlying mechanisms for efficacy and resistance remains a major focus in ICI research. In this Research Topic, we have included several recent studies investigating ICI biomarkers. For example, Zhang et al. reviewed that IL-37 suppresses tumors by regulating macrophage polarization and inhibiting VEGF, which correlates with better survival, while IL-38 promotes progression by reducing CD8^+^ T cell recruitment and upregulating PD-1/PD-L1. Xie et al. verified that the CONUT score can effectively assess the prognosis of NSCLC patients and may apply to ICI treatment scenarios. Liu et al. described a case of advanced NSCLC that developed an acquired SDC4-ROS1 fusion after multi-line therapy with ICI, suggesting that this fusion may be a potential resistance-related marker.

Adverse events remain a critical concern in ICI therapy, requiring effective prediction and monitoring strategies. Cai et al. found that the systemic immune-inflammatory index (SII) reliably predicts severe ICI-related pneumonitis (CIP) (AUC = 0.81), with severe CIP associated with shorter survival (7.23 vs. 22.17 months). Mariniello et al. reported a case of ICI-associated myocarditis-myositis-myasthenia overlap, emphasizing the need for early monitoring and multidisciplinary intervention. Zhu et al. also reported a case of ICI-related type 1 diabetic ketoacidosis, highlighting the importance of glucose/C-peptide monitoring and collaborative care.

Together, these contributions illuminate the complex interplay between the tumor, its microenvironment, and the host immune system. Spanning from mechanistic insights to clinically accessible tools, these studies offer valuable insights for predicting response, managing resistance, and early detecting life-threatening toxicities.

## Rare subtypes and novel strategies

The treatment of rare NSCLC subtypes has always been a challenge. Thus, in this Research Topic, we also collected several cases of ICI therapy for rare NSCLC subtypes. For example, Guo et al. reported a KRAS-mutated pulmonary enteric adenocarcinoma (PEAC) case with limited response to standard ICI-chemo and colorectal-focused chemo, highlighting the need for subtype-specific therapies. Zhang et al. described a stage I pulmonary pleomorphic carcinoma (PPC) with rapid postoperative recurrence; subsequent chemo-ICI-antiangiogenic combination therapy achieved 9-month PFS, suggesting ICIs as a new therapeutic option for this subtype.

Meanwhile, ICIs may also be effective in NSCLC when combined with other malignancies. For instance, Sussman et al. reported on a patient with concurrent resectable stage IIIA NSCLC and PDGFRA-rearranged myeloid neoplasm. Neoadjuvant chemotherapy plus nivolumab, alongside imatinib for the myeloid neoplasm, led to a pathological complete response (pCR) in the pulmonary lesions, with no recurrence over 12-month follow-up.

Additionally, novel combination therapies hold therapeutic potential. Liu et al. reported that indacaterol inhibits NSCLC cell growth via GLUT1/MCT4 metabolic pathways and synergizes with PD-L1 inhibitors in preclinical models. Notably, concurrent medication timing may be critical: Wang et al. found that baseline glucocorticoid (GC) use was superior to early GC use, indicating timing-dependent GC-ICI synergy.

These studies suggest that the principles of immunotherapy can be extended to challenging rare subtypes and even to patients with synchronous malignancies. They also highlight the potential of innovative combination strategies to enhance efficacy and may broaden the horizon for ICI therapy.

## Conclusion

This Research Topic presents key advances in ICI therapy for NSCLC, covering resectable, unresectable stage III, and advanced settings. It addresses critical challenges, including efficacy in rare subtypes, identification of predictive biomarkers, and strategies for monitoring ICI-related toxicities. These studies also highlight the value of personalized approaches, while novel combination therapies and insights into treatment timing offer new directions.

Building on these findings, future studies should focus on validating novel biomarkers in diverse populations, clarifying primary and acquired resistance mechanisms using multi-omics strategies, and optimizing rational combination therapies in clinical trials. These steps may help refine personalized ICI treatment and ultimately improve outcomes for NSCLC patients across all stages and subtypes.
